# Saudi specialty certificate in endodontics: program directors perceptions of candidate selection

**DOI:** 10.3389/fmed.2026.1915523

**Published:** 2026-09-30

**Authors:** Ali Robaian AlQahtani, Yazeed AlQahtani, Shahad AlBader, Abdullah AlShehri, Mohammed A. S. Abuelqomsan, Abdulaziz Abdulwahed, Ahmed AlMokhatieb, Mubashir Baig Mirza

**Affiliations:** 1 Conservative Dental Sciences, College of Dentistry, Prince Sattam Bin Abdulaziz University, Al-Kharj, Saudi Arabia; 2 Dental Student, College of Dentistry, Prince Sattam Bin Abdulaziz University, Al-Kharj, Saudi Arabia

**Keywords:** dental education, endodontics, program directors, residency, Saudi Arabia

## Abstract

**Introduction:**

Program Directors (PDs) evaluate a range of factors when selecting candidates. Understanding their perspectives may help applicants better prepare for the selection process. This study explored PDs' perspectives on selection criteria for the Saudi Specialty Certificate in Endodontics (SSE) program and examined whether these perspectives differed according to gender, experience, hospital setting, and geographic region.

**Methods:**

This study aimed at an attempted census of all identifiable current and former SSE PDs registered with SCFHS. A previously validated survey with 30 questions covering seven domains: GPA, Exams, Research, Services and Electives, Interview, Recommendations, and Miscellaneous was distributed to the PDs via the Saudi Commission for Health Specialties (SCFHS) platform to ensure anonymity. Data collection spanned four months. Descriptive statistics, along with independent sample t-test and ANOVA, were used for analysis.

**Results:**

32 PDs responded, yielding a response rate of 55.17%. Most PDs were current (53.1%), male (81.2%), from the central region (50%), affiliated with the Ministry of Health (34.4%), and had over three years of experience (43.8%). PDs prioritized the services and electives (mean 4.04 ± 0.87) and recommendations (mean 3.97 ± 0.60) domains. Key factors influencing these preferences included clinical competency and staff reputation (mean 4.37 ± 0.87), participation in endodontic electives (mean 4.28 ± 0.81), service residency experience (mean 4.28 ± 0.88), and professional attire (mean 4.28 ± 0.85), among others. Exams (mean 3.31 ± 1.02) and research (mean 3.40 ± 0.98) were less prioritized, mainly due to low emphasis on publication output (mean 2.96 ± 1.44). Statistical analysis revealed no significant differences across most subgroup comparisons. An exploratory difference was observed in the recommendation domain according to hospital setting (*p* = 0.034).

**Conclusion:**

The PDs' reported priorities indicate that services and electives have the highest descriptive mean, with candidates' reputation and performance during that period being the most significant factors. Although opinions differed across hospital settings, the findings should be interpreted cautiously and considered exploratory.

## 1 Introduction

Endodontics is a branch of dentistry that focuses on the knowledge and treatment of diseases affecting the dental pulp and periapical tissues (1). It employs a multidisciplinary approach, drawing insights from various fields, including microbiology, oral biology, pathology, epidemiology, radiology, and materials science, among others (2). Additionally, it increasingly overlaps with advanced fields such as tissue engineering and genetic research and has seen significant developments in diagnostic and therapeutic standards for pulpal and periapical diseases (3). While recent advances have made treatment more predictable, endodontics remains highly challenging and requires profound knowledge and clinical expertise (1). As educational and clinical training vary between institutions and countries, establishing robust academic programs and advanced training opportunities is imperative (4, 5).

In Saudi Arabia, the Saudi Commission for Health Specialties (SCFHS) oversees and accredits all health specialties, including dentistry. Candidates seeking postgraduate studies in Dentistry must, at the time of application, have completed a five-year Bachelor's degree in Dentistry and either finished or be nearing completion of a mandatory one-year internship and pass the Saudi Dental Licensing Exam (SDLE). Postgraduate education generally comprises a three-year Master's program emphasizing theoretical knowledge, research, and clinical skills across six specialties: Endodontics, Restorative Dentistry, Orthodontics, Prosthodontics, Periodontics, and Pediatric Dentistry. Alternatively, candidates may pursue a more competitive, clinically oriented Board program lasting three to five years, covering specialties such as Endodontics, Restorative Dentistry, Orthodontics, Prosthodontics, Periodontics, Oral and Maxillofacial Surgery, Oral Medicine and Pathology, Pediatric Dentistry, and Family Dentistry (6). By comparison in the United States, following the completion of a four-year Doctor of Dental Surgery (DDS) program, graduates can pursue advanced dental education recognized by the American Dental Association (ADA) in nine specialties, including Oral and Maxillofacial Surgery, Orthodontics and Dentofacial Orthopedics, Periodontics, Endodontics, Prosthodontics, Oral and Maxillofacial Pathology, Oral and Maxillofacial Radiology, Pediatric Dentistry, or opt for one- to two-year advanced general dentistry residency programs such as Advanced Education in General Dentistry (AEGD) or General Practice Residency (GPR) (7).

As of 2023, restorative dentistry had the highest number of residents, followed by orthodontics and endodontics. This trend may be attributed to students' interest or the availability of training opportunities in the area (6). The Saudi Board of Endodontics (SBE), now recognized as the Saudi Specialty Certificate in Endodontics (SSE), was established by the SCFHS in 2006 as a four-year residency program aiming to provide trainees with in-depth knowledge and clinical skills. It uses a rigorous curriculum that combines didactic and clinical training through lectures, literature reviews, seminars, quizzes, clinical practice, and case presentations (8, 9). In 2019, SCFHS implemented significant changes to the SSE program to improve healthcare standards and meet the growing number of residents and training centers, in line with Vision 2030 in KSA. It is now a three-year, full-time course conducted at the SCFHS-accredited institution without external rotations, like other benchmark programs in North America and Europe. The training has also changed from being a time and requirement-based system to a more competency-based education (CBE), as dictated by the Canadian Medical Association's CanMEDS framework (10).

Given the high prevalence of dental caries in KSA and the increasing number of teeth requiring root canal treatment (RCT), demand for trained specialists in this field is growing (11). According to a 2016 survey, there were 410 active endodontists in Saudi Arabia, with 197 more enrolled in postgraduate courses, mostly the SSE program. Currently, 28 approved centers in Saudi Arabia and 1 in Dubai conduct the SSE program. Most of these centers are in the major cities of Riyadh and Jeddah. For the selection of the students, various factors are considered, including Grade Point Average (GPA), performance on the SDLE, undergraduate course grades, any previous or ongoing research activities, publications, letters of recommendation, demonstrated interest in endodontics, private practice experience, English language proficiency, and results from the personal interview. Despite Saudi students' reported enthusiasm for postgraduate education, the SCFHS website does not clearly state the selection criteria, though it describes program entry requirements and curriculum details (12, 13).

Program directors (PDs) are central to recruiting and selecting candidates, and these processes can vary significantly across institutions because of personal preferences and differing priorities that shape how candidates are assessed. This lack of standardization may lead to inconsistencies in candidate selection, thereby creating a stressful experience for the applicants (6). In Saudi Arabia, three previous studies on PDs' perceptions of candidate selection for dental board programs have been conducted: periodontics, orthodontics, and restorative dentistry (14–16). These studies highlighted critical insights for prospective candidates. Similarly, studies in the United States across various dental specialties, including orthodontics, periodontics, and endodontics, have been conducted (17–19). Notably, this study represents the first on endodontic PDs in the region and only the second of its kind worldwide.

Given the limited research available, particularly concerning the factors influencing candidate selection processes for SSE within the Kingdom of Saudi Arabia, this study aims to provide a thorough exploration of PDs' decision-making. These insights are essential for optimizing student preparedness and improving their chances of selection. Additionally, the research aims to evaluate PDs' perspectives across various demographic variables, thereby identifying potential differences in opinions that may influence candidate selection.

## 2 Materials and methods

### 2.1 Study design and ethical approval

This cross-sectional study involved PDs recruited from various centers registered with SCFHS. This study was carried out in accordance with the Declaration of Helsinki. The Standing Committee for Bioethics Research at Prince Sattam bin Abdulaziz University granted ethical approval under protocol number SCBR-422/2025, issued on February 9, 2025. Survey permissions were requested through the Tawasol platform (request number 589795) on the SCFHS website on February 15, 2025. In accordance with the bioethics clearance, an informed consent statement was obtained from each participant, and only those who acknowledged participation were directed to the survey. The statement emphasized maintaining anonymity and assured that no identifiable information was collected.

### 2.2 Survey instrument and data collection

A secure, web-based survey was distributed via Qualtrics (Qualtrics LLC, Utah, USA) to 58 PDs registered with the SCFHS who were involved in the SSE program, either currently or previously. The questionnaire was adapted from a previously validated survey assessing residency selection perspectives (14). Before deployment, it was pilot-tested for clarity and relevance to endodontics residency selection. Three experienced endodontic faculty members (AA, AAM, and MASA) assessed the questionnaire for clarity, relevance, and suitability before implementation. Feedback from this review contributed to refining the instrument, including removing one SDLE-related item deemed inapplicable because specialty-specific SDLE scores were unavailable. The same instrument was subsequently used in another study on assessing candidate selection criteria for the Saudi Restorative Dentistry Board Program (16). The data collection process lasted for four months, starting from February 15 to June 15, 2025. During the first two months, 19 responses were collected. An automated reminder email was sent on April 16, 2025, after two months, and 13 more responses were collected. Because the population was small and clearly defined, an attempted census approach was used, in which all identifiable current and former PDs were invited to participate in the survey.

### 2.3 Questionnaire

At the start of the questionnaire, the first part sought participant consent, recorded as “yes” or “no.” If the answer was “no,” the survey would terminate with a message of gratitude for taking the time to respond. Participants who responded “yes” were taken to the core questions of the questionnaire, organized into three distinct sections. The first section included five questions designed to gather demographic information, including gender, current professional status, years of experience in the role, geographical region of practice, and the type of hospital environment in which the participant works.

The second section comprised fifteen inquiries, designed to evaluate how candidate-specific performance metrics influence admission outcomes. The first domain included metrics such as GPA, recognition through awards, the prestige of the graduating institution, and placement on the Dean's Honorary List. The second domain included SDLE examination scores and successful completion of international licensing examinations, such as the INBDE. The next domain comprised six questions that examined the candidates' research through both quantitative and qualitative metrics, the prestige of the journals that published their work, the degree of alignment with endodontic research, and any oral or poster presentations they may have delivered. The fourth domain evaluated the impact of candidate interviews on the overall assessment. In this domain, three parameters were assessed: the weight of the interview relative to GPA and SDLE, choice of professional attire, and endodontic knowledge.

The next section included fifteen questions, six of which focused on the Service and elective domain. These included participating in endodontic electives during internships, working as an endodontic service resident, taking electives at the same institute, pursuing international electives, and electives under a renowned endodontist. The domain recommendations included several important aspects: the need for multiple letters, letters from specialized endodontists, the recommending dentist's reputation, the mode of sending the recommendation, and the letter's quality and content. Lastly, the miscellaneous domain included two queries related to English-language proficiency and involvement in community service.

The 30 questions listed in Tables 1, 2 aimed at understanding PDs' preferences regarding candidate selection. All items were measured on a five-point Likert scale to assess their significance, which included 1 for strongly disagree, 2 for disagree, 3 for neither agree nor disagree, 4 for agree, and 5 for strongly agree.

**Table 1:** Means and standard deviations of GPA, exams, research, and interviews domains.

Domains with individual queries	Strongly Disagree Freq (%)	Disagree Freq (%)	Neither Agree/Disagree Freq (%)	Agree Freq (%)	Strongly Agree Freq (%)	Mean ± SD
I. GPA						**3.52** ± **0.87**
1. Does the grade point average (GPA) improve the chance of getting accepted?	3 (9.4)	1 (3.1)	10 (31.3)	9 (28.1)	9 (28.1)	3.62 ± 1.21
2. Does being on the Dean's honorary list improve the chance of acceptance?	3 (9.4)	5 (15.6)	9 (28.1)	8 (25)	7 (21.9)	3.34 ± 1.26
3. Do candidates who gained awards or honors have a better chance of getting accepted in your program?	2 (6.3)	4 (12.5)	9 (28.1)	13 (40.6)	4 (12.5)	3.40 ± 1.07
4. Does the reputation of the graduated dental school influence their chance of acceptance into your program?	4 (12.5)	2 (6.3)	3 (9.4)	12 (37.5)	11 (34.4)	3.75 ± 1.34
II. Exams						**3.31** ± **1.02**
5. Is the Saudi Dental License Exam (SDLE) score important?	2 (6.3)	5 (15.6)	7 (21.9)	11 (34.4)	7 (21.9)	3.5 ± 1.19
6. Does a candidate who passed international licensing examinations, such as INBDE, have a better chance of being accepted into your program?	4 (12.5)	6 (18.8)	8 (25)	10 (31.3)	4 (12.5)	3.12 ± 1.23
III. Research						**3.40** ± **0.98**
7. Is having a general experience in research important?	2 (6.3)	4 (12.5)	3 (9.4)	16 (50)	7 (21.9)	3.68 ± 1.14
8. Is the quantity of publications important?	6 (18.8)	8 (25)	6 (18.8)	5 (15.6)	7 (21.9)	2.96 ± 1.44
9. Is the quality of publications important?	3 (9.4)	4 (12.5)	4 (12.5)	12 (37.5)	9 (28.1)	3.62 ± 1.28
10. Is publishing in prestigious journals important?	4 (12.5)	6 (18.8)	5 (15.6)	13 (40.6)	4 (12.5)	3.21 ± 1.26
11. Are publications in Endodontics the most important?	5 (15.6)	5 (15.6)	2 (6.3)	12 (37.5)	8 (25)	3.40 ± 1.43
12. Does presenting posters or oral presentations at events improve the chance of acceptance?	2 (6.3)	2 (6.3)	7 (21.9)	18 (56.3)	3 (9.4)	3.56 ± 0.98
IV. Interview						**3.86** ± **0.68**
13. Does the interview carry the same weight as the SDLE score and the GPA?	2 (6.3)	10 (31.3)	1 (3.1)	9 (28.1)	10 (31.3)	3.46 ± 1.39
14. Do you prefer candidates who are well-dressed during the interview?	0	2 (6.3)	2 (6.3)	13 (40.6)	15 (46.9)	4.28 ± 0.85
15. Do you look for the level of knowledge in Endodontics during the interview?	2 (6.3)	1 (3.1)	5 (15.6)	16 (50)	8 (25)	3.84 ± 1.05

Likert scale: Strongly disagree 1; Disagree 2; Neither agree nor disagree 3; Agree 4; Strongly agree 5. Values in bold indicate the overall Mean and Standard deviations for that particular domain.

**Table 2:** Means and standard deviations of services and electives, recommendations and miscellaneous domains.

Domains with individual queries	Strongly Disagree Freq (%)	Disagree Freq (%)	Neither Agree/Disagree Freq (%)	Agree Freq (%)	Strongly Agree Freq (%)	Mean ± SD
V. Services and Electives						**4.04** ± **0.62**
16. Is taking Endodontics elective rotations during internship important?	0	0	7 (21.9)	9 (28.1)	16 (50)	4.28 ± 0.81
17. Working as “service resident” improves the chance of accepting an applicant?	1 (3.1)	0	3 (9.4)	13 (40.6)	15 (46.9)	4.28 ± 0.88
18. Candidates who worked/took electives in your department have a better chance of getting accepted?	1 (3.1)	0	7 (21.9)	13 (40.6)	11 (34.4)	4.03 ± 0.93
19. Does the reputation and performance (during clinics or between staff) influence their chance of acceptance?	1 (3.1)	0	2 (6.3)	12 (37.5)	17 (53.1)	4.37 ± 0.87
20. Do candidates who have worked/took electives abroad have a better chance of getting accepted?	3 (9.4)	2 (6.3)	12 (37.5)	9 (28.1)	6 (18.8)	3.4 ± 1.16
21. Do the candidates who worked/took electives with a distinguished Endodontist have a better chance of acceptance?	1 (3.1)	1 (3.1)	6 (18.8)	15 (46.9)	9 (28.1)	3.93 ± 0.94
VI Recommendations						**3.97** ± **0.60**
22. Is having a recommendation letter important?	0	2 (6.3)	9 (28.1)	13 (40.6)	8 (25)	3.84 ± 0.88
23. Is having multiple recommendations important?	2 (6.3)	2 (6.3)	8 (25)	10 (31.3)	10 (31.3)	3.75 ± 1.16
24. Are recommendations from an Endodontist important?	0	2 (6.3)	3 (9.4)	16 (50)	11 (34.4)	4.12 ± 0.83
25. Is personally knowing the recommending person important?	1 (3.1)	2 (6.3)	1 (3.1)	14 (43.8)	14 (43.8)	4.18 ± 0.99
26. Is the reputation of the recommending person important?	0	1 (3.1)	4 (12.5)	15 (46.9)	12 (37.5)	4.18 ± 0.78
27. Is the mode of recommendation, such as phone calls or written letters, important?	3 (9.4)	2 (6.3)	5 (15.6)	12 (37.5)	10 (31.3)	3.75 ± 1.24
28. Is the quality of the language and content of the recommendation important?	0	2 (6.3)	8 (25)	10 (31.3)	12 (37.5)	4 ± 0.95
VII Miscellaneous						**3.71** ± **0.64**
29. Does participating in community service activities and volunteering improve the chance of acceptance?	3 (9.4)	4 (12.5)	10 (31.3)	12 (37.5)	3 (9.4)	3.25 ± 1.10
30. Is being fluent in English important?	0	2 (6.3)	0	20 (62.5)	10 (31.3)	4.18 ± 0.73

Likert scale: Strongly disagree 1; Disagree 2; Neither agree nor disagree 3; Agree 4; Strongly agree 5. Values in bold indicate the overall mean and standard deviation for each domain.

### 2.4 Statistical analysis

Data were analyzed using SPSS version 23 (Chicago, IL, USA), employing a two-phase approach to assess the study variables. The data were subjected to descriptive analysis to present the sample's demographic characteristics. The mean and standard deviation were calculated for numerical variables. A binary scale was used to determine the level of importance of the variables, with positive responses (“yes”: Agree, Strongly Agree) and negative responses (“no”: Neutral, Disagree, Strongly Disagree). Mean and Standard Deviation for the different factors were calculated for various demographic characteristics, including region, experience, gender, and hospital type. Normality was assessed using the Shapiro–Wilk test, which indicated no significant departures from normality in any domain and supported parametric analyses. An independent-samples t-test at *p* ≤ 0.05 was conducted to examine gender differences. ANOVA was conducted to identify statistically significant differences among participants' experiences, geographic region, and hospital type at *p* ≤ 0.05.

## 3 Results

### 3.1 PDs' demographics

Of the 58 recipients of the link, 32 responded, yielding a response rate of 55.17%. Among the respondents, 53.1% were current PDs, while 46.9% were former PDs. Demographically, 81.2% identified as male and 18.8% as female. Experience varied: 43.8% had over three years of experience, 18.8% were first-time PDs, and 37.4% had between 1 and 3 years of experience. Geographically, half were from the central region, 25% from the west, 12.5% from the south, and 9.4% from the east, with only one PD from the north. Hospital affiliation included 34.4% from the Ministry of Health, 28.1% from universities, 28.1% from military hospitals, 6.3% from private hospitals, and 3.1% from general hospitals, as illustrated in Figure 1.

**Figure 1:**
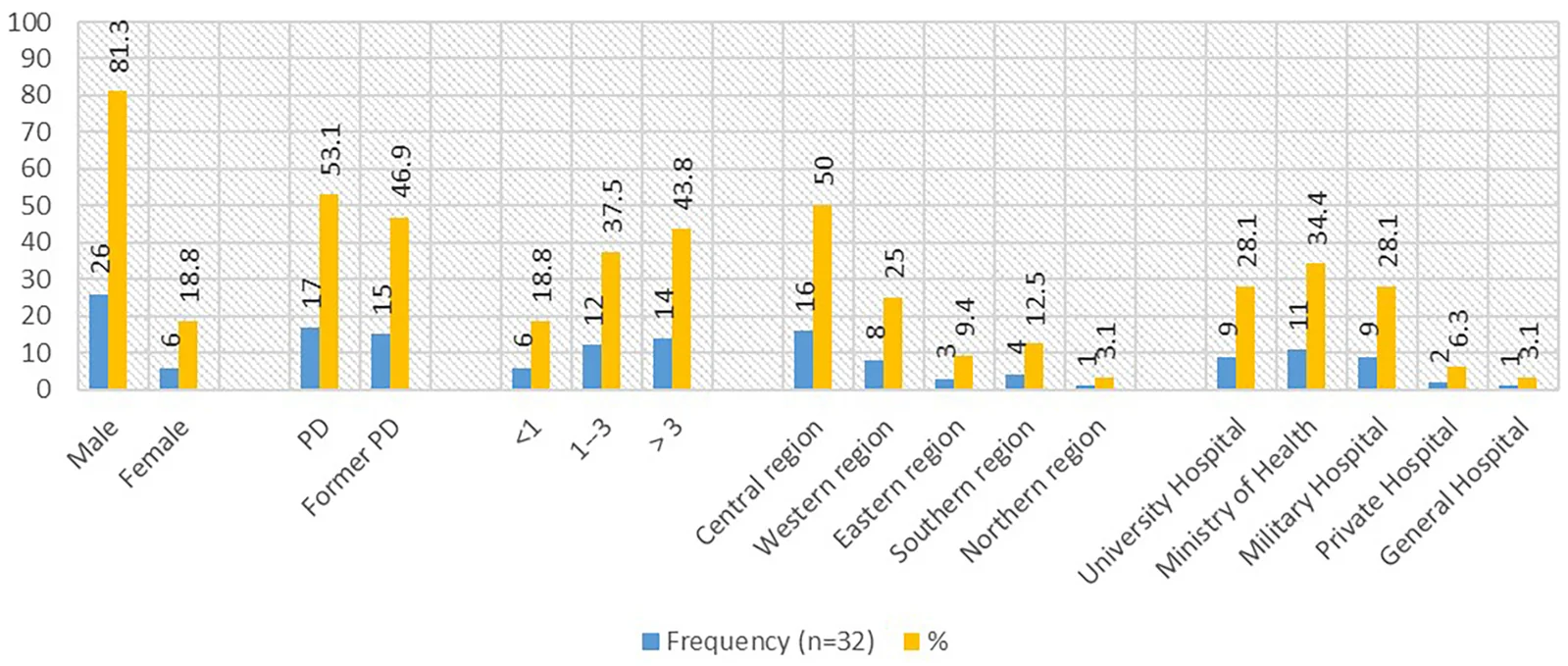
Program director demographics.

### 3.2 PDs' response based on individual queries

The PDs placed greater emphasis on candidates' reputation among staff and their performance during electives, with an average score of 4.37 ± 0.87. Moreover, taking endodontic electives during internship and serving as a service resident both received higher average scores of 4.28 ± 0.81 and 4.28 ± 0.88, respectively. Additionally, being well-dressed for the interview positively influenced the PDs' perceptions. In contrast, the number of publications by candidates received the least weight, with an average score of 2.96 ± 1.44. This was followed by the importance assigned to candidates who had passed international licensing exams such as the INBDE, with a score of 3.12 ± 1.23. Additionally, publications in reputable journals and involvement in community service were not particularly appealing to the PDs, with scores of 3.21 ± 1.26 and 3.25 ± 1.10, respectively, as seen in Tables 1, 2.

### 3.3 PDs' response based on domains

Among the evaluated domains, service and electives scored highest, with a mean of 4.04 ± 0.62, indicating that respondents prioritized this factor over others. The recommendation domain was rated next, with a mean of 3.97 ± 0.60. The interview domain followed, with a mean score of 3.86 ± 0.68. Conversely, the exams area was the least prioritized by PDs, with a mean score of 3.31 ± 1.02, while the research domain also received a relatively low mean score of 3.40 ± 0.98, as shown in Tables 1, 2.

### 3.4 PDs' response based on gender

The independent-samples t-test showed no statistically significant differences across any study domain. Both male and female PDs preferred services and electives, with mean scores of 4.03 ± 0.67 and 4.14 ± 0.43, respectively. Interestingly, males placed a higher priority on the recommendation domain (4.01 ± 0.59), whereas females showed a slightly greater inclination toward interviews (4.00 ± 0.67). Conversely, both groups reported Exams and Research as their least favored areas, with average scores of 3.39 ± 1.06 for males and 3.00 ± 0.84 for females in exams, and a mean of 3.4 for research shared by both genders, as detailed in Table 3.

**Table 3:** Means and standard deviations of PD responses based on their gender.

Domain	Gender	n	Mean	Std. dev	t (df)	*p*-value	95% CI	Cohen's d
GPA	Male	26	3.52	0.91	0.16 (30)	0.874	−0.74, 0.60	−0.08
	Female	6	3.58	0.74				
Exams	Male	26	3.39	1.06	0.83 (30)	0.415	−0.24, 1.02	0.39
	Female	6	3.00	0.84				
Research	Male	26	3.41	1.04	0.01 (30)	0.989	−0.64, 0.64	0.00
	Female	6	3.42	0.74				
Interview	Male	26	3.83	0.70	0.53 (30)	0.600	−0.77, 0.45	−0.23
	Female	6	4.00	0.67				
Service and Electives	Male	26	4.03	0.67	0.37 (30)	0.711	−0.59, 0.37	−0.18
	Female	6	4.14	0.43				
Recommendation	Male	26	4.01	0.59	0.53 (30)	0.599	−0.46, 0.76	0.24
	Female	6	3.86	0.73				
Miscellaneous	Male	26	3.69	0.65	0.48 (30)	0.638	−0.77, 0.47	−0.23
	Female	6	3.83	0.68				

n, total number of participants; t (df), independent sample t test with degree of freedom; *p*-value with significance at ≤0.05; CI, 95% Confidence Interval; Cohen's d, effect size.

### 3.5 PDs' response based on experience

ANOVA showed variation in responses among PDs with varying experience levels; however, these differences were not statistically significant, as shown in Table 4. Nonetheless, among the newer PDs with less than one year of experience, recommendations (mean 4.25 ± 0.35) and services or electives (mean 4.13 ± 0.56) are prioritized, while domains such as research and interviews are least emphasized, with means of 3.77 ± 0.98 and 3.83 ± 0.83, respectively. Among PDs with 1–3 years of experience, the most valued domains were services and electives (mean 4.05 ± 0.69) and recommendations (mean 3.91 ± 0.65), while domains such as examinations (mean 3.12 ± 0.88) and research (mean 3.17 ± 0.94) received the lowest scores. Experienced PDs with over 3 years of experience focus more on services and electives (mean 4.01 ± 0.62) and recommendations (mean 3.9 ± 0.65), while domains like exams (mean 3.21 ± 1.15) and research (mean 3.44 ± 1.02) are less emphasized, as seen in Table 4.

**Table 4:** Means and standard deviations of PD responses based on their experience.

Domains	Experience	*n*	Mean	Std. dev	F (df1, df2)	*p*-value
GPA	0–1 years	6	4.03	0.74	1.44 (2, 29)	0.250
	1–3 years	12	3.31	0.61		
	>3 years	14	3.49	1.05		
Exams	0–1 years	6	3.91	0.86	1.35 (2, 29)	0.276
	1–3 years	12	3.12	0.88		
	>3 years	14	3.21	1.15		
Research	0–1 years	6	3.77	0.98	0.76 (2, 29)	0.481
	1–3 years	12	3.17	0.94		
	>3 years	14	3.44	1.02		
Interview	0–1 years	6	3.83	0.83	0.01 (2, 29)	0.990
	1–3 years	12	3.86	0.67		
	>3 years	14	3.87	0.68		
Service and Electives	0–1 years	6	4.13	0.56	0.07 (2, 29)	0.921
	1–3 years	12	4.05	0.69		
	>3 years	14	4.01	0.62		
Recommendation	0–1 years	6	4.25	0.35	0.79 (2, 29)	0.462
	1–3 years	12	3.91	0.65		
	>3 years	14	3.90	0.65		
Miscellaneous	0–1 years	6	3.91	0.66	0.38 (2, 29)	0.699
	1–3 years	12	3.70	0.72		
	>3 years	14	3.63	0.60		

*n*, total number of participants; F (df1, df2), Analysis of variance (ANOVA) with numerator and denominator degrees of freedom; *p*-value with significance at <0.05.

### 3.6 PDs' response based on the hospital setting

University hospital PDs placed higher priority on recommendation-related factors (mean 4.26 ± 0.38) and the lowest on the examination domain (mean 2.83 ± 1.09). Meanwhile, Ministry of Health (MOH) PDs placed greater emphasis on the services and electives domain (mean 4.07 ± 0.43) and rely least on exams (mean 3.18 ± 0.75). In military hospitals, PDs emphasize interviews (mean 4.03 ± 0.85) but tend to downplay the importance of research (mean 3.44 ± 1.21). Private hospital PDs rank miscellaneous factors highest (mean 4.25 ± 0.35) and place the least emphasis on research (mean 2.91 ± 1.29), as illustrated in Table 4. A one-way ANOVA demonstrates a significant difference among University, the Ministry of Health (MOH), and military PDs in the recommendation domain, F (2,26) = 3.87, *p* = 0.034. Tukey *post hoc* showed that university-based PDs assigned significantly higher recommendation scores than MOH PDs, whereas military PDs did not differ significantly from either group, as seen in Table 5. However, because data from private and general hospitals were excluded from inferential analysis due to insufficient sample sizes for reliable statistical comparisons, the findings should be interpreted as exploratory.

**Table 5:** Means and standard deviations of PD responses based on their hospital setting.

Domains	Hospital setting	*n*	Mean	Std. dev	F (df1, df2)	*p*-value
GPA	University	9	3.44	0.94	0.02 (2, 26)	0.978
	MOH	11	3.56	0.75		
	Military	9	3.47	1.12		
Exams	University	9	2.83	1.09	1.23 (2, 26)	0.309
	MOH	11	3.18	0.75		
	Military	9	3.77	1.22		
Research	University	9	3.31	1.05	0.03 (2, 26)	0.973
	MOH	11	3.44	0.74		
	Military	9	3.44	1.21		
Interview	University	9	3.84	0.81	0.82 (2, 26)	0.452
	MOH	11	3.75	0.44		
	Military	9	4.03	0.85		
Service and Electives	University	9	4.08	0.94	0.05 (2, 26)	0.955
	MOH	11	4.07	0.43		
	Military	9	3.94	0.55		
Recommendation	University	9	4.26^a^	0.38	3.87 (2, 26)	0.034*
	MOH	11	3.89^b^	0.73		
	Military	9	3.96^ab^	0.42		
Miscellaneous	University	9	3.72	0.56	1.52 (2, 26)	0.239
	MOH	11	3.72	0.68		
	Military	9	3.50	0.70		

*n*, total number of participants; F (df1, df2), Analysis of variance (ANOVA) with numerator and denominator degrees of freedom; *p*-value with significance at <0.05; *, statistically significant difference; Superscripts ^a^ and ^b^ indicate significant differences, and ^ab^ is a non-significant difference seen by the Tukey *post-hoc* test.

### 3.7 PDs' response based on the region

PD preferences varied across geographic regions, highlighting differences in opinion; however, these variations were not statistically significant. PDs from the central region reported an average preference score of 3.98 ± 0.59, while those from the southern region had a higher average of 4.5 ± 0.36 in the domain services and electives. In the western region, PDs often relied on recommendations (mean 4.19 ± 0.50), whereas PDs in the eastern region placed greater emphasis on GPA (mean 4.58 ± 0.28). PDs in the central (mean 3.5 ± 0.93), western (mean 2.62 ± 0.69), and eastern regions (mean 3.83 ± 1.15) viewed examinations as the least important selection criterion. Additionally, PDs from the south seemed to prioritize research less, with an average score of 3.04 ± 1.48, as shown in Table 6. The response from the PD in the northern region (*n* = 1) was excluded because of the inadequate sample size. Subgroup comparisons should be considered exploratory since some categories have small sample sizes.

**Table 6:** Means and standard deviations of PD responses based on region.

Domains	Region	*n*	Mean	Std. Deviation	F (df1, df2)	*p*-value
GPA	Central	16	3.62	0.81	2.45 (3, 27)	0.084
	West	8	3.15	0.68		
	East	3	4.58	0.28		
	Southern	4	3.18	1.31		
Exams	Central	16	3.50	0.93	1.85 (3, 27)	0.159
	West	8	2.62	0.69		
	East	3	3.83	1.15		
	Southern	4	3.62	1.60		
Research	Central	16	3.60	1.00	1.96 (3, 27)	0.138
	West	8	2.87	0.51		
	East	3	4.21	0.63		
	Southern	4	3.04	1.48		
Interview	Central	16	3.87	0.75	0.97 (3, 27)	0.434
	West	8	3.58	0.55		
	East	3	4.21	0.83		
	Southern	4	4.16	0.57		
Service and Electives	Central	16	3.98	0.59	1.01 (3, 27)	0.407
	West	8	3.87	0.83		
	East	3	4.21	0.34		
	Southern	4	4.50	0.36		
Recommendation	Central	16	3.84	0.59	1.20 (3, 27)	0.324
	West	8	4.19	0.50		
	East	3	4.23	0.57		
	Southern	4	4.25	0.27		
Miscellaneous	Central	16	3.77	0.44	1.08 (3, 27)	0.393
	West	8	3.43	0.90		
	East	3	4.16	1.04		
	Southern	4	3.62	0.47		

*n*, total number of participants; F (df1, df2), Analysis of variance (ANOVA) with numerator and denominator degrees of freedom; *p*-value with significance at <0.05.

## 4 Discussion

The objective of residency programs is to select competent residents who will evolve into successful practitioners. However, the lack of measurable metrics aligned with PDs' expectations, coupled with ambiguity in selection criteria, poses challenges for prospective candidates (18). This study aims to provide insights into the SSE program selection process from PDs' perspective. Although previous research has addressed selection criteria in different programs, this research is unique because it focuses specifically on endodontic PDs in Saudi Arabia. Because selection criteria differ across dental specialties, results from PDs in other specialties cannot be considered universal for the endodontic discipline. The results indicate that PDs valued the services/elective and recommendation domains. Specifically, aspects related to the candidate's skills and reputation among staff, completing endodontic electives, and being a service resident were more important. Conversely, examination and research activities were comparatively less important in the selection process, particularly the importance of INBDE and the quantity of published research. Furthermore, there were no significant differences in responses of PDs concerning different domains based on their gender, experience in years, or geographical location. Although differences were observed in the recommendation domain across hospital settings, these findings should be interpreted cautiously and considered exploratory given the limited subgroup sample sizes and multiple subgroup comparisons performed.

The Board programs in Saudi Arabia evaluate candidates using percentage-based grading. The SDLE exam score accounts for 55% of the assessment, while GPA accounts for 30%. The remaining 15% includes research, clinical services, specialized dental electives, and community volunteering, as well as additional factors such as Letters of Recommendation and interviews, which PDs assess directly (20). GPA is often cited as a selection criterion in global educational settings and has been shown to be correlated with future academic success (21). However, it cannot be the sole criterion, as this study reveals that endodontic PDs place significantly more importance on the reputation of the graduating dental school than on GPA itself, similar to orthodontic PDs in Saudi Arabia and endodontic PDs in the US, who considered the prestige of the dental school to be an influential determinant (15, 19, 22). In contrast, studies on periodontal PDs in Saudi Arabia and the U.S. show that GPA influences evaluations more than other academic factors such as institutional prestige, being on the Dean's honor list, or awards (14, 18). This trend was also observed among pediatric and orthopedic PDs in Saudi Arabia, and U.S. plastic surgery PDs also report paying less attention to school rankings when choosing candidates (23–25). However, prestigious institutions often leverage standardized assessments, which enhance the evaluation of student competencies and may lead to more skilled graduates (26).

The examination domain received the least attention from PDs in this study. INBDE is recognized as a rigorous standard in dentistry and is known for its comprehensive assessment of student knowledge (18). However, PDs in this study tend to prioritize SDLE results over INBDE scores, which aligns with findings among PDs in Saudi Arabia (14, 15). A reasonable explanation is that the SSE is more clinically oriented and involves practical clinical work that requires local licensure, such as the SDLE. In the US, PDs primarily rely on interviews and clinical bench tests for decision-making because the INBDE uses a pass/fail system. Instead, they emphasize qualitative factors such as letters of recommendation, personal statements, clinical grades, and institutional evaluations (27). The lower scores in these domains in this study likely reflect PDs' understanding that both GPA and exams are established criteria and that all applicants are understood to have met those requirements at the time of application (14).

Research is a key factor in selecting candidates for academic programs, with recent research suggesting that research experience distinguishes high-performing graduates (28). Prior research experience appears crucial, as indicated by PDs' average score of 3.68 ± 1.14, similar to reports from PDs in the Saudi Periodontics program (14). Conversely, US periodontic PDs placed less emphasis on this aspect (18). While metrics such as publication volume are relevant and may indicate visibility, they do not necessarily reflect a candidate's intellectual merit or scientific quality (29). According to this study's findings, PDs in the Kingdom often overlook this aspect and instead focus on research quality and scientific presentation, which highlight a candidate's preparation, dedication, and enthusiasm (30). Periodontic PDs in the Kingdom similarly suggest a 25% approval for research quantity (14).

The results show that over half of the PDs regard interviews as equally important as SDLE scores and GPA. This perspective aligns with the opinions of periodontic PDs from both Saudi Arabia and the USA (14, 18). A previous study on selecting undergraduate dental students found that interviews were an essential part of the selection process; however, they did not correlate with academic achievement (19, 31). PDs in charge of urology and plastic surgery programs in Saudi Arabia regarded interviews as the most significant factor because they could interact directly with candidates (22, 32). Another study suggested that interviews should be structured and validated against specific performance attributes to be effective (33). While details about the interview processes used by the PDs remain undisclosed, and data on the consistency of these interviews across centers in the Kingdom are lacking, future guidelines should incorporate this aspect. Dressing appropriately on the day of the interview significantly influences interviewers' perceptions, as indicated by a high mean score of 4.28 among participating PDs in this study, similar to those reported for restorative and pediatric PDs in Saudi Arabia (16, 23). This finding supports existing literature on recruitment, especially during the initial stages of the interview, which shows that a person's attire plays a critical role in shaping assessments of professionalism and competence (34, 35).

In this research, PDs gave the highest priority to services and electives, as indicated by the mean score of 4.04, which surpasses the scores given by other domains. Candidates' performance and reputation in electives are important to PDs in this research, consistent with studies on Saudi periodontics and pediatric PDs (14, 23). As SSE is an advanced program, students with strong clinical skills are more likely to influence PDs. Additionally, soft skills are equally important, such as having professional behavior and a good reputation among patients, peers, and instructors. This helps build trust and create a conducive environment for treatment (36). Participation as a service resident and having taken endodontic electives during internship influence PDs' opinions. Electives are considered crucial in dentistry, as they prepare students for clinical practice and offer skills that complement their theoretical knowledge (37). PDs in this study have nearly equal preferences for candidates who take electives at the same institution or with a distinguished endodontist, consistent with results from Saudi periodontic PDs (14). However, fewer than half of PDs have a positive attitude toward students who took electives overseas because of their familiarity with the domestic program's curriculum and rigor (38).

In general, recommendations play an important role in candidate selection as reflected in the responses of PDs in this study. Studies on applications to the United States Medical Licensing Examination (USMLE) and microsurgical residency found that recommendation letters were a key factor in evaluating candidates (39, 40). Similarly, Laskin et al. found that up to 90% of PDs believed they gained insights from personalized recommendations rather than relying on standard referral forms when selecting candidates for Oral Surgery residency programs (41). Fewer letters, rather than multiple letters, and letters from more reputable individuals appealed to orthodontic and pediatric PDs, similar to our findings (17, 23). Additionally, orthodontic PDs preferred letters of recommendation from university-based faculty members over those from other hospitals, while oral surgery PDs preferred letters from within their fraternity over those from non-oral surgeons or institute Deans (17, 41). Among other factors, English proficiency impressed the endodontic PDs in this study, similar to previous studies in Saudi Arabia on restorative and pediatric PDs (16, 23). In contrast, community service, an important part of healthcare program requirements, had less impact on endodontic PDs.

Most participants in this study belonged to the Central and Western parts of the kingdom, which aligns with previous Saudi studies examining regional perspectives (14, 16). It could also be related to the presence of the two largest cities in Saudi Arabia, which host the highest number of accredited centers. In this study, PDs new to their roles preferred recommendations more than more experienced PDs, similar to a comparable study on Saudi pediatric residency selection (23). In contrast, Saudi periodontic PDs in their study reported that more experienced PDs relied more on recommendations and trusted their peers (14). Regarding the type of hospital setting, PDs from Universities relied on recommendations more frequently than their counterparts from non-academic hospitals. The observed variation may reflect differences in institutional culture and training environments within the Saudi healthcare system. This contrasts with the results reported from the Saudi Urology candidate selection study (42). Nonetheless, differing opinions may be attributed to the fact that university-based residency programs are often closely integrated with academic activities. Consequently, recommendation-related information may provide additional insights into applicants' academic potential. However, these findings should be interpreted cautiously, as the study assessed reported perceptions rather than actual selection decisions, and the subgroup analyses were exploratory.

This is the first study to examine perspectives of endodontic PDs in Saudi Arabia, considering factors such as experience, gender, hospital type, and region. The findings will help PDs and other relevant parties enhance the selection process for future reference. The findings are expected to influence candidates and help them organize and prepare their applications more effectively. Despite the study's comprehensive nature, it has several limitations. The response rate was 55.17%; non-response bias cannot be excluded, and the findings may not be fully generalizable to all eligible current and former SSE PDs. Additionally, the absence of participation from PDs in the northern region, as well as from PDs affiliated with private and general hospitals, further constrains the generalizability of the findings. Although these subgroups were not included in the analysis, the limited statistical power of some subgroups means that these findings should be regarded as exploratory. In addition, multiple subgroup comparisons were performed, and no formal adjustment for multiplicity was applied. Therefore, statistically significant findings should be interpreted cautiously and considered hypothesis-generating. Additionally, some queries encompassed multiple constructs and may be considered double-barreled. Furthermore, the questionnaire assesses multiple distinct domains rather than a single construct, thereby limiting the validity of internal consistency estimates. Although several academic backgrounds were evaluated, the questionnaire specifically did not assess the influence of previous postgraduate degrees or other advanced academic qualifications. Moreover, self-reporting bias cannot be ruled out completely in a survey-based investigation. Further studies need to incorporate a structured assessment tool and quantitative criteria for candidate assessment.

## 5 Conclusion

This study provides insights into PDs perspectives on candidate selection criteria for the SSE program; however, it reflects their reported priorities and should not be interpreted as evidence that these factors independently predict admission outcomes or future resident performance. The services and electives domain had the highest descriptive means, with queries related to reputation among staff and performance during electives receiving the highest ratings, while exams and research were less prioritized. Although variations based on PD demographics were observed, only the recommendations domain showed an exploratory difference by hospital setting. However, given the exploratory nature of the subgroup analyses and multiple comparisons performed, this finding should be interpreted cautiously and considered hypothesis-generating.

## Data Availability

The original contributions presented in the study are included in the article/Supplementary Material, further inquiries can be directed to the corresponding authors.
